# Emetine in the Dysentery of Children

**Published:** 1914-07-11

**Authors:** 


### Emetine in the Dysentery of Children.

In the *Journal of Tropical Medicine and Hygiene* Captain R. G. Archibald, pathologist to the Wellcome Research Laboratories, Khartoum, deals with the treatment of acute amœbic dysentery in children. He gives notes of cases in which he tried the emetine method of Professor (now Sir) Leonard Rogers with success. Although he does not endorse all that Rogers has claimed for this treatment, he feels justified in advancing that: (1) Young children are extremely tolerant of emetine. In severe cases of entamœbic dysentery it is advisable to commence with an initial dose of 1-6 gr. for a child of two, and to repeat this dose every twelve hours until $\frac{1}{2}$ gr. has been given. (2) The total amount of emetine administered should be controlled by the evidence obtained by microscopical examination of the stools, a procedure which should also be carried out at intervals during convalescence. (3) In order to avert relapses, the continued treatment by emetine after the patient's apparent recovery from dysentery is advisable. (4) In entamœbic dysentery of the Sudan emetine may be required in larger doses than are usually employed in other countries. He adds, further, the advice that in tropical countries the fæces of all natives should be examined before taking them into employment. This, we fear, is a counsel of perfection; and even if the principle were adopted, it would not suffice to abolish the risks of infection. For an individual who passed the test successfully one week might acquire infectivity the next, and to repeat the test once a month or once a week would be prohibitive from the expense. However, Captain Archibald's paper is very valuable from the clinical side.

